# Repeat AngioVac Debulking of a Large Right Atrial Thrombus With Extension From the Hepatic Vein

**DOI:** 10.1155/cric/9155374

**Published:** 2025-04-22

**Authors:** Leonard Palatnic, Tim Avino, Edward J. Spangenthal, David M. Zlotnick

**Affiliations:** ^1^Department of Medicine, University at Buffalo, Jacobs School of Medicine and Biomedical Sciences, Buffalo, New York, USA; ^2^Department of Medicine, Roswell Park Comprehensive Cancer Center, Buffalo, New York, USA

## Abstract

The AngioVac mechanical aspiration system has emerged in the literature as a viable treatment option for patients with intracardiac masses and thrombi and who are deemed high risk for cardiac surgery. Notably, it has been shown to be an effective treatment modality for the debulking of thrombi in the setting of malignancy. We present a case of repeat AngioVac debulking of a large right atrial thrombus with extension from the hepatic vein in the setting of advanced malignancy complicated by *Enterococcus faecium* endocarditis.

## 1. Introduction

The AngioVac mechanical aspiration system has emerged in the literature as a promising treatment modality for percutaneous debulking of intracardiac masses and thrombi in patients deemed to be too high risk for cardiac surgery [1]. Its utility has been demonstrated by the Registry of AngioVac Procedures in Detail (RAPID) study, which prospectively analyzed 234 patients who underwent AngioVac treatment between 2016 and 2019, with 113 of these patients being treated for right heart masses [2]. The study illustrated that 70%–100% mass or thrombus removal was achieved in more than 73% of patients, while only 36 procedure-related complications were reported, along with a single reported death [2]. The RAPID registry illustrated that the AngioVac mechanical aspiration system is a safe and effective tool for removing vascular thrombi and cardiac masses. Since the publication of the RAPID registry, retrospective analyses, such as Katapadi et al. and Mullins et al., have provided additional evidence supporting the use of the AngioVac system for percutaneous thrombectomy [3, 4]. This technique has been increasingly adopted in clinical practice, particularly in high-risk patients who may not be suitable candidates for conventional surgical interventions. We present a case of repeat AngioVac debulking of a large right atrial thrombus with extension from the hepatic vein in the setting of advanced malignancy complicated by *Enterococcus faecium* endocarditis.

## 2. Case Presentation

A 50-year-old female with past medical history of Stage IV colorectal adenocarcinoma with metastasis to the liver status post exploratory laparotomy with low anterior resection, paroxysmal atrial fibrillation, and provoked pulmonary embolism on anticoagulation presented for planned exploratory laparotomy, partial hepatectomy, and percutaneous laser ablations of residual liver metastases. Postoperatively, the patient acutely developed shock and was found to have *Enterococcus faecium* bacteremia. A transesophageal echocardiogram (TEE) revealed a large thrombus in the inferior vena cava (IVC) extending to the right atrium. CT further characterized the mass as a nonocclusive thrombus within the left hepatic vein extending into the IVC and right atrium. Following discussion with a multidisciplinary heart team, the patient was deemed to be high risk for cardiac surgery and was referred for thrombectomy with the AngioVac system.

**Figure 1:**
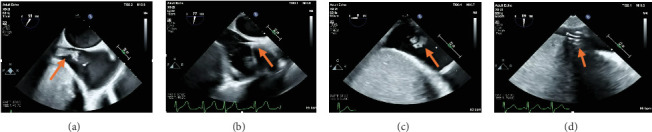
Intraprocedural transesophageal imaging displaying (a) right atrial thrombus extending from the IVC (orange arrow). (b) AngioVac debulking of IVC thrombus (orange arrow). (c) Hepatic vein thrombus (orange arrow). (d) AngioVac debulking of hepatic vein thrombus (orange arrow).

**Figure 2:**
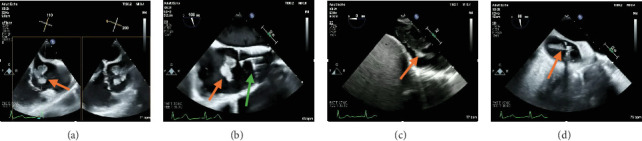
Intraprocedural transesophageal imaging displaying (a) recurrent right atrial thrombus extending from IVC (orange arrow). (b) AngioVac debulking (green arrow) of right atrial thrombus (orange arrow). (c) IVC thrombus extending from hepatic vein (orange arrow). (d) AngioVac thrombectomy of hepatic vein (orange arrow).

As such, the patient was brought to the cardiac catheterization lab. An AngioVac C20 catheter was advanced into the right atrium, and debulking of the thrombus was performed under TEE guidance, with 100% debulking appreciated (Figure 1). Debulking then proceeded into the IVC, where 100% debulking was appreciated (Figure 1). Debulking then proceeded into the proximal hepatic vein (Figure 1). Additional thrombus was visualized on TEE in the distal hepatic vein; however, the C20 was too big to debulk in this area. A Penumbra 16-Fr lightening catheter was advanced into the hepatic vein, and additional thrombus debulking was performed. A small persistent residual thrombus in the distal hepatic vein was noted; however, > 90% debulking was appreciated. Final IVC venography was performed and demonstrated complete patency of the IVC into the right atrium and proximal hepatic vein, at which time the decision was made to end the procedure. The patient's blood pressure, heart rate, and oxygen saturation level remained stable throughout the procedure. Pathology was consistent with fibrinoid necrotic mass. A follow-up CT revealed interval clearance of thrombus. Patient's blood cultures continued to be positive, and repeat TTE 10 days later revealed a newly formed large right atrial thrombus with evidence of extension from the hepatic vein. As the residual thrombus in the hepatic vein was felt to be a nidus for ongoing infection and thrombus formation, the patient was again deemed a candidate for thrombectomy followed by left lateral hepatectomy. Debulking began with the AngioVac C20 catheter in the right atrium proceeding into the IVC and proximal hepatic vein (Figure 2). Significant reduction in vegetative burden was noted (> 90%). The AngioVac cannula was then removed. IVC venography demonstrated complete patency of the IVC into the right atrium. The patient's blood pressure, heart rate, and oxygen saturation level remained stable throughout the procedure. Pathology was consistent with an atrial thrombus.

Subsequently, the patient underwent left hepatectomy 24 h later. Her blood cultures cleared, and she was ultimately discharged home. Repeat MRI imaging throughout her postoperative course demonstrated no new thrombus formation.

## 3. Discussion

We present a case of repeat debulking with mechanical thrombectomy of a large right atrial thrombus with extension from the hepatic vein in the setting of advanced malignancy complicated by *Enterococcus faecium* endocarditis. Currently, only the European Society of Cardiology guidelines for infective endocarditis recognize percutaneous debulking of right intra-atrial masses as a treatment option, having been given a Class IIb indication [5]. There have been case reports of AngioVac debulking of intracardiac thrombus in the setting of malignancy reported in the literature [6–8]. Lebehn et al. described the successful use of AngioVac debulking of a large mobile mass in the right atrium in a patient with metastatic pancreatic adenocarcinoma and deemed too high risk for cardiac surgery [4]. Similar to our case, Lebehn et al. display the benefit of the AngioVac mechanical aspiration system in both therapeutic and diagnostic utility and, as such, demonstrate that it is a valuable treatment modality that may serve as an effective alternative to surgery.

Patients with symptomatic right-sided intracardiac masses and/or thrombi as well as patients with large vegetations (> 2 cm) and who are deemed to be too high risk for surgery may be suitable candidates for percutaneous debulking due to the greater risk for embolization.

Additionally, patients who are considered too high risk for surgery yet continue to experience persistent bacteremia or recurrent septic emboli despite appropriate medical therapy may qualify as potential candidates for percutaneous debulking. Finally, similar to the case presented by Lebehn et al., our case demonstrates that the use of the AngioVac mechanical aspiration system can assist in diagnosis through histopathological analysis. In these patient populations, the primary goals of debulking include symptomatic relief, enhancing antibiotic penetration by reducing vegetative burden, achieving source control, and minimizing the risk of embolization.

There are risks associated with the use of the AngioVac mechanical aspiration system. Due to the need for large bore access, common complications may include access site bleeding, localized bleeding, and overt bleeding requiring blood transfusion. Additional complications include incomplete thrombus debulking, right ventricular free wall perforation, and embolization to the pulmonary arteries [1]. In the RAPID registry, the most common complications associated with AngioVac debulking were hemorrhage (3.8%), trauma at cannula insertion site (3.4%), and distal embolization (3.0%) [2]. Notably, there was one procedure-related death described in the registry, secondary to intraoperative embolization of a caval tumor thrombus resulting in massive pulmonary embolism [2].

It is important to note that in order to perform the procedure, it is essential to have a perfusionist or specialist proficient in using an extracorporeal pump. Additionally, having a physician with advanced cardiac imaging training involved in the procedure is beneficial, as they can assist in visualizing the intracardiac mass and/or thrombi with intraprocedural imaging to guide the procedure and evaluate the success of the thrombectomy. Finally, given the potential complications associated with the procedure, operators with substantial experience are advised to use the AngioVac mechanical aspiration system to ensure effective debulking and reduce the risk of complications. We recommend a multidisciplinary heart team approach to evaluating and treating these patients.

## 4. Conclusion

To conclude, in patients with advanced malignancy complicated by the development of infectious thrombi, cardiac surgery is often not a viable option due to associated perioperative risk. We present a unique case of repeat AngioVac debulking of a large right atrial thrombus with extension from the hepatic vein in the setting of Stage IV colorectal adenocarcinoma with liver metastasis and persistent bacteremia. Minimally invasive thrombectomy/debulking with the AngioVac system appears to be a safe and effective alternative to cardiac surgery for the management of high-risk patients with right-sided intracardiac thrombus.

## Data Availability

All data, including laboratory values, imaging files, and other raw data, were obtained from the patient's medical records and can be made available in a deidentified format.
